# The Golden Fountain - Is urine the miracle drug no one told you about?

**Published:** 2010-05-25

**Authors:** Loeffler Jutta M

**Affiliations:** 1 University College London, London, UK

**Keywords:** Urine therapy

## Editorial

Drinking or local application of human or animal urine for medicinal purposes has been practiced all over the world for millennia. Documented prescriptions in Europe originate from ancient Egypt, Greece and Rome. While many of the advances of antique medicine were forgotten after the fall of the Roman Empire, the use of urine and other excrements enjoyed continued popularity in mediaeval times. Ancient Indian yogic texts and ancient Chinese documents describe benefits of drinking one’s own urine, and it can be assumed that people in Africa, the Americas and other parts of the world have traditionally used urine for various medical indications for a very long time, too.

The almost 100,000 hits of the search for “urine therapy” on Google, and the over 150 videos on the subject on YouTube are an indicator that drinking “waters out of thine own cistern” is still, or again, rather popular today.

The supposed indications for urine therapy, ancient or contemporary, are too numerous to recite. There is, it seems, virtually nothing urine won’t cure. Modern proponents use pseudoscience to explain the benefits of the various, mostly exaggerated, components of urine. Some hint at a conspiracy by the medical establishment and the pharmaceutical industry to keep the knowledge of the many fantastic healing properties of cheaply available urine a secret. There is no money to be made from urine, well, unless one was to write a book about its many virtues. But, seriously, what do we really know? Urine is mostly water, lots of urea (25g/d) and uric acid (1g/d), creatinine (1.5g), various electrolytes (10g/d, mostly NaCl), phosphate and organic acids (3g/d), only trace amounts of proteins (40-80mg/d, most of which are albumin, and only insignificant amounts of antibodies or enzymes), varying traces of (not necessarily active) hormones, glucose and water-soluble vitamins. Urea has a known potent diuretic effect which is at the base of the 19^th^ century application of cow’s urine in the Apozème Suisse for oedema or ascites. Urine is sterile where it is produced in the kidney, but once it has left the body, it is usually contaminated. It is not toxic per se. There may be rare situations where urine is the cleanest liquid at hand to pour over a dirty wound, or the only liquid to drink when buried under a collapsed building or lost at sea for days, but most of the time there are better or tastier ways to improve one’s health.

This said, the situation described in the paper by Ogunshe, Fawole and Ajayi in this journal [1], is quite different. Here, the use of human or cows’ urine does not stem from an esoteric search for eternal youth or someone’s personal rage against the establishment, but from bare necessity in an economically struggling part of the world where modern medicine or the money to pay for it, is lacking. The authors describe the administration of urine to babies and young children with febrile and other convulsions, as a traditional therapy that may be gaining popularity because of increasing poverty. They examine the aspect of bacterial contamination and antibiotic resistance in samples of children’s and cows’ urine. Contamination and bacterial growth in concoctions containing urine is an important issue in a warm climate and when the treated patient is a fragile infant. Unsurprisingly, Ogunshe et al. recovered the usual urinary suspects, but more importantly, they found high resistance rates against the more commonly available antibiotics. In the absence of proper clinical trials it is difficult to prove that traditional urine therapy contributes to childhood mortality in Nigeria, but given the results of the present study, the treatment of vulnerable and already ill children with urine should be strongly discouraged. First, do no harm.
